# Canine Visceral Leishmaniasis in Boyer Ahmad District, Kohgiluyeh & Boyer Ahmad Province, Southwest of Iran

**Published:** 2012

**Authors:** A Moshfe, M Mohebali, E Afshoun, A Mousavizadeh, Z Zarei, H Abidi, B Akhoundi, V Barati, S Joukar

**Affiliations:** 1 Cellular and Molecular Research Center, Yasuj University of Medical Sciences, Yasuj, Iran; 2 Dept. of Medical Parasitology & Mycology, School of Public Health, Tehran University of Medical Sciences, Tehran, Iran; 3 Center for Research of Endemic Parasites in Iran (CREPI), Tehran University of Medical Sciences, Tehran, Iran; 4 Research Management, Yasuj University of Medical Sciences, Yasuj, Iran; 5 Meshkin-Shahr research station, National Institute of Health Research, Tehran University of Medical Sciences, Tehran, Iran; 6 School of Medicine, Yasuj University of Medical Sciences, Yasuj, Iran

**Keywords:** Visceral leishmaniasis, Direct agglutination test, Ownership dogs, Iran

## Abstract

**Background:**

Mediterranean type of visceral leishmaniasis (VL) is present in different parts of Iran. Several studies have identified dogs as the main reservoirs of the VL caused by *Leishmania infantum* in Iran and other Mediterranean regions. This study aimed to determine the seroprevalence of canine visceral leishmaniasis as animal reservoir host for human visceral leishmaniasis in Boyer Ahmad district in southwest of Iran.

**Methods:**

A seroepidemiological study was carried out to determine the seroprevalence of canine visceral leishmaniasis (CVL) among ownership dogs by using direct agglutination test (DAT) in 23 of 182 villages of Boyer Ahmad district, during August 2009 to August 2010. One hundred and seventy serum samples from ownership dogs were selected by multi-stage cluster sampling in villages of Boyer Ahmad district. All samples were tested by DAT and anti-*Leishmania* antibodies titers at ≥ 1:320 was considered as positive.

**Results:**

Of the 170 serum samples, 10% were positive by DAT at titers of 1:320 and higher. No statistical significant difference was found between male (10.7%) and female (8.3%) seroprevalence. The highest seroprevalence rate (15.1%) was observed among the ownership dogs of four to seven years age. Altogether, seventeen (25.4%) of the seropositive dogs had clinical signs and symptoms.

**Conclusion:**

It seems that Boyer Ahmad district is an endemic area for canine visceral leishmaniasis in Iran.

## Introduction

Every year, approximately 500,000 new cases of visceral leishmaniasis (VL) which cause 59,000 human deaths annually, are reported from various parts of the world and incidence rate of the disease is increasing in some countries (1, 2).

Since 1996, human cases of VL have been reported from Kohgilouye & Boyer Ahmad Province, mostly, from Boyer Ahmad district (3, 4). It seems that kala-azar is going to distribute in this area. The seroprevalence rate of infection was reported 3.1% in children lived in Boyer Ahmad district (5). Our knowledge about canine visceral leishmaniasis (CVL) is highly important for control of human visceral leishmaniasis in endemic areas.

Domestic dogs (*Canis familiaris*) are the most important animal reservoir hosts of CVL, which is transmitted among canines and to humans by phlebotomine sand flies (6–8). Domestic dog is considered as an important risk factor for human infections in the endemic areas of the disease in Iran (9).

CVL caused by *L. infantum* is an endemic zoonotic disease in the Mediterranean basin and Middle East, including Iran where seroprevalence rate of disease has been reported from 10 to 37% (7–14).

As the high proportion of infected dogs is asymptomatic, therefore, detection of specific antibodies remains the method of choice for mass screening of dogs in epidemiological surveys and evaluation of prevalence (9, 10, 12).

Serological methods are highly sensitive and non-invasive, so they are appropriate tools for the determination of VL infection in field conditions (15, 16). Several diagnostic tests are available to detect anti-*Leishmania* antibodies in canine sera. In the present study, the direct agglutination test (DAT) was used as sero-diagnostic tool, because it is a simple as well as valid test and does not require specialized equipments (17).

This study aimed to determine the seroprevalence of CVL in various parts of Boyer Ahmad district to more identifying the role of dog as natural reservoir of human kala-azar in the areas to presenting effective control program of human VL to health authorities.

## Materials and Methods

### Study area

Boyer Ahmad district is located in Kohgiluyeh & Boyer Ahmad Province, southwest of Iran. The city of Boyer Ahmad is situated at an altitude of 1490 m above sea level and is closed to the Dena high mountains. The weather of this district is moderate to cold mountainous. Its population is estimated to be 169967 among which 42% was settled in urban areas and 58% in rural areas. Out of this a part belongs to nomad tribes.

### Sampling

This descriptive and cross sectional study was conducted in Boyer Ahmad district. The investigation was carried out over a period of 13 months from August 2009 to August 2010 on 170 ownership dogs. Our Sampling method was multi stage cluster sampling. Twenty three villages (cluster) from 243 villages were selected randomly and serum samples were randomly taken from domestic dogs in each cluster based on the population of dogs. All the selected dogs were physically examined. Dog age was determined by interviewing dog owners. Blood samples were taken from the selected dogs by venapuncture in villages, poured into 10 ml polypropylene tubes and processed 4-10 h after collection. The collected blood samples were centrifuged at 800 ×g for 5-10 min, and the separated sera were stored at -20°C. All the serum samples were tested by DAT in the Parasitology Laboratory in the School of Medicine, Yasuj University of Medical Sciences.

### Direct Agglutination Test

The *Leishmani infantum* antigens for this study were prepared in the leishmaniasis Laboratory, Department of Medical Parasitology and Mycology, School of Public Health, Tehran University of Medical Sciences, Iran. The principal phases of the procedure for making DAT antigen were mass production of promastigotes of *L. infantum* (MCAN/IR/07/Moheb-gh) in RPMI1640 plus 10% fetal bovine serum, tripsinization of the parasites, staining with Coomassie brilliant blue and fixing with formaldehyde 2% (16, 17).

The dog serum samples were tested by DAT, initially, for screening purposes; dilutions were made 1:80 and 1:320. Samples with titers 1:320 were diluted further to end-point titer 1:20480. Negative control, wells (antigen only; on each plate) and known negative and positive control serum samples were tested in each plate daily.

The cut off titer was defined as the highest dilution at which agglutination was still visible, as blue dot, compared with negative control wells, which had clear blue dots. The positive standard control serum prepared from dogs with *L*. *infantum* infection (at 1:20480) in an endemic area and confirmed by microscopy, culture and animal inoculation. Quantitative results obtained with DAT were expressed as an antibody titer, i.e. the reciprocal of the highest dilution at which agglutination (large diffuse blue mats) is still visible after 18 h incubation at room temperature (16). Two individuals read the tests independently. The cut off value was determined in previous studies by experimental infection (7–9).

We considered anti-*Leishmania* antibodies titers at ≥ 1:320 as canine visceral *Leishmania* infection in this investigation.

### Data analysis

Chi-square and Fisher exact tests were used to compare seroprevalence values relative to gender, age and clinical signs. Analyses were conducted using SPSS software version 13.5, with a probability (*P*) value of <0.05 as statistically significant.

## Results

The sero-prevalence rate (SPR) in titers 1:320 and above was 10%. Seventy (25.4%) of the seropositive dogs showed at least one clinical sign including skin lesions, such as exfoliative dermatitis and ulcerations, local or generalized lymphadenopathy, cachexia, low appetite, alopecia, ocular lesions, epistaxis and lameness. No clinical signs and symptoms were seen in 50 (74.6%) of seropositive dogs. Anti-*Leishmania* antibodies, using the cut-off value of 1:320 and above were detected in male and female domestic dogs. The seroprevalence values among male and female animals were 10.7% and 8.3%, respectively (Table 1). No statistically significant differences were observed between canine *Leishmania* infection and gender (*P*= 0.781).

**Table 1:** Sero-prevalence of canine *Leishmania* infection by gender in Boyer Ahmad district, Southwest of Iran (2009-2010)

Gender	No of dogs (%)	DAT negative No. (%)	DAT positive ≥ 1:320	

			No.	Seroprevalence (%)
Male	122 (71.8)	109 (71.2)	13	10.7
Female	48 (28.2)	44 (28.8)	4	8.3
Total	170 (100)	153 (100)	17	10

X^2^= 0.206 df= 1 *P*= 0.781

Table 2 shows that 26.9% of symptomatic dogs were seropositive whereas 6.9% of asymptomatic dogs were sero- positive. Significant differences were observed between the canine *Leishmania* infection and clinical symptoms (*P*= 0.006).

**Table 2:** Sero-prevalence of canine *Leishmania* infection by signs and symptoms in Boyer Ahmad district, Southwest of Iran (2009-2010)

Signs& Symptoms	No of dogs (%)	DAT negative No. (%)	DAT positive ≥ 1:320	

			No.	Seroprevalence (%)
positive*	26 (15.3)	19 (12.4)	7	26.9
Negative	144 (84.7)	134 (87.6)	10	6.9
Total	170 (100)	153 (100)	17	10

* Dogs showed at least one clinical sign including skin lesions, such as exfoliative dermatitis and ulcerations, local or generalized lymphadenopathy, cachexia, low appetite, alopecia, ocular lesions, epistaxis and lameness X^2^= 9.767 df= 1 *P*= 0.006

Considering the animal age groups, the highest seroprevalence rate (15.1%) was found in dogs with 4-7 years old (Table 3). No statistically significant differences were observed between canine *Leishmania* infection and age groups.

**Table 3:** Sero-prevalence of canine *Leishmania* infection by age in ownership dogs in Boyer Ahmad district (2009-2010)

Age group (yr)	No of dogs (%)	DAT positive ≥ 1:320	

		No.	Seroprevalence (%)
0-3	75 (44.1)	4	5.3
4-7	73 (42.9)	11	15.1
> 7	22 (13)	2	9.1
Total	170 (100)	17	10

X^2^= 4.466 df= 2 *P*= 0.107

Of the 170 dogs, 26 (15.3%) had at least one clinical sign and 73.1% of them did not have titer of antibody while 26.9% had antibody titer detected by DAT (Table 4). The titers of antibody in symptomatic dogs were at 1:1280 to 1:20480 levels.

**Table 4:** Distribution of titers of anti *Leishmania* antibodies in symptomatic and asymptomatic ownership dogs by DAT in Boyer Ahmad district (2009-2010)

Titer of Antibody	No of dogs (%)	Symptomatic		Asymptomatic	

		No.	%	No.	%
< 1:80	146 (85.9)	19	13	127	87
1:80	6 (3.5)	0	0	6	100
1:160	1 (0.6)	0	0	1	100
1:320	2 (1.2)	0	0	2	100
1:640	4 (2.4)	0	0	4	100
1:1280	1 (0.6)	1	100	0	0
1:2560	4 (2.4)	3	75	1	25
1:20480	6 (3.5)	3	50	3	50
Total	170 (100)	26	15.3	144	84.7

## Discussion

Dogs and wild canines are animal reservoir hosts for *L. infantum* in both old and new worlds (6–8). Determination of the prevalence of canine *Leishmania* infection is necessary to define control measures for zoonotic visceral leishmaniasis (18).

The studies in the last decade showed that *L. infantum* was the principal agent of the disease in human and animal reservoirs in different parts of Iran (6, 19, 20). Dogs and wild carnivores such as jackals, foxes and wolves, which have been found, infected with *L. infantum* (21, 22) and domestic dogs are considered the most important source and reservoirs of *L. infantum* infection particularly in the endemic areas of Iran (7, 9, 14).

Visceral leishmaniasis, caused by *L. infantum*, is endemic in some part of Iran such as north western and southern regions (6, 16, 23). No survey on CVL has been carried out in Boyer Ahmad district in two last decades thus; this study was conducted in this area.

For canine leishmaniasis, serological test is considered as a sensitive and useful technique and is well correlated with clinical signs. According to previous studies (15, 17, 24, 25) the performance of the DAT for detection of *L. infantum* infection in humans and dogs was desirable. Therefore, we used DAT for the determination of sero-prevalence of canine *Leishmania* infection.

Based on our results, seroprevalence of CVL in Boyer Ahmad was determined as 10% using the cut-off value of 1:320 and above. Based on a new study that designed for VL seroprevalence determination in Iran, seropositivity rate of CVL was 8.3% (9).

No statistical differences were found between *Leishmania* infection and gender in our study. Similar results were found by Abranches et al. in Portugal; Pozio et al. in Italy; Sideris et al. in Greece, Bokai et al.; Mohebali et al. and Moshfe et al. in Iran (7, 8, 11, 26–28).

In the current study, we found canine *Leishmania* infection mostly in dogs with 4-7 years old. No statistical differences were found between age groups and *Leishmania* infection. Our previous study in northwest of Iran, revealed a greater seroprevalence rate in older dogs, indicating that the probability of exposure to the bite of sand flies infected with *L. infantum* increases with the age of infected dogs (26, 29). But in Boyer Ahmad district it seems that the disease has a new distribution, as the new cases have been reported.

The high prevalence of *Leishmania* infection in dogs appears to be due to high exposure with *Leishmania* parasites both in villages and in the wild areas.

However, the role of asymptomatic but seropositive dogs (10 out of 17) is difficult to explain without a follow-up study. In our previous study, the asymptomatic infected dogs were found to be a source of *L. infantum* infection. Undoubtedly, this condition indicates previous contact with the parasite, but we do not know whether these dogs gained a proper immunity or whether they will subsequently develop the disease (30).

Leishmaniasis should be considered in patients presenting with a compatible clinical symptom and a history of travel to an endemic area, even if this occurred several months or years ago. Appropriate counseling should be provided to travelers, military personnel, researchers, and other groups of travelers who may be exposed to sand flies in endemic areas. Early detection and treatment of visceral leishmaniasis can reduce the incidence of severe illness and death. It is important for clinicians to change their perceptions of the population at risk.

## Conclusion

The most important result was a proportion of seropositivity for leishmaniasis (6.9%) among dogs without clinical signs of canine leishmaniasis. These data are very important because ownership dogs can play an important role in the epidemiology of this zoonotic disease. Furthermore, the domestic dog population could be helpful sentinels to follow the progress of the disease in endemic areas.

Control programs on infected dogs will be almost impossible without taking effective measures to determine the status of seropositive in asymptomatic dogs. Essentially, elimination of infected animals has been recommended (31), but alternative control measures should be recommended for ethical and social reasons.
